# Supplementary material for: Rational Optimization of Cathode Composites for Sulfide-Based All-Solid-State Batteries
Source: Nanomaterials (Basel). 2023 Jan 12;13(2):327. doi: 10.3390/nano13020327 (PMC9866434; doi:10.3390/nano13020327)
Supplement: Supplementary file 1 [file nanomaterials-13-00327-s001.zip › nanomaterials-2146751-supplementary.pdf]

Supplementary Information

Rational Optimization of Cathode Composites for Sulfide-Based All-Solid-State Batteries

Artur Tron *, Raad Hamid, Ningxin Zhang and Alexander Beutl *

AIT Austrian Institute of Technology GmbH, Center for Low-Emission Transport, Battery Technologies, Giefinggasse 2, 1210 Vienna, Austria

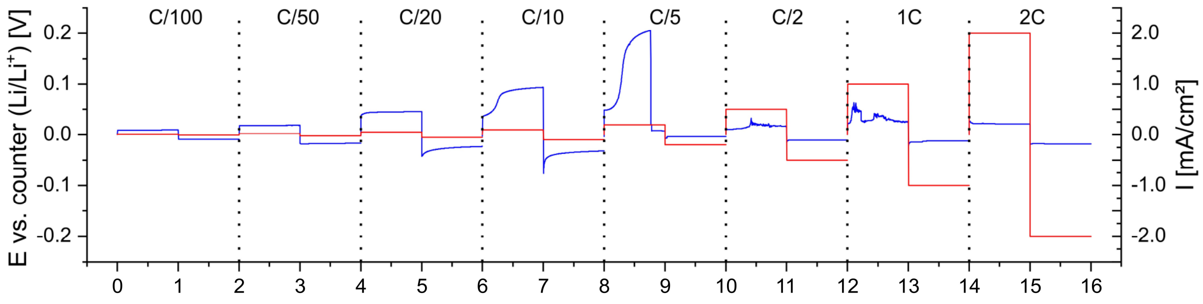

Figure S1. critical current density of Li6PS5Cl electrolyte membrane. A current density of 1C corresponds to 1 mA/cm². The measurement was conducted by sandwiching a 300 µm thick electrolyte membrane in-between two Li electrodes of 16 mm diameter and applying 10 MPa of pressure.

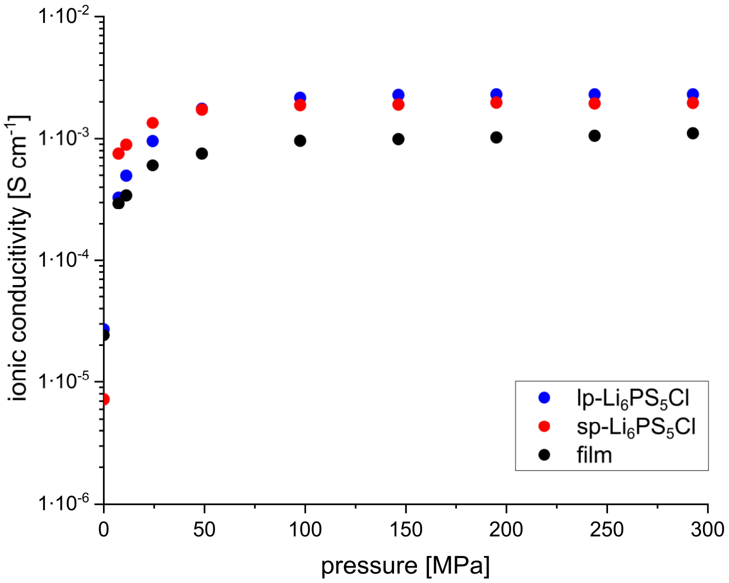

Figure S2. ionic conductivity vs. pressure plots for powder pellets using lp-, sp-Li_6PS_5Cl, and the prepared electrolyte films.

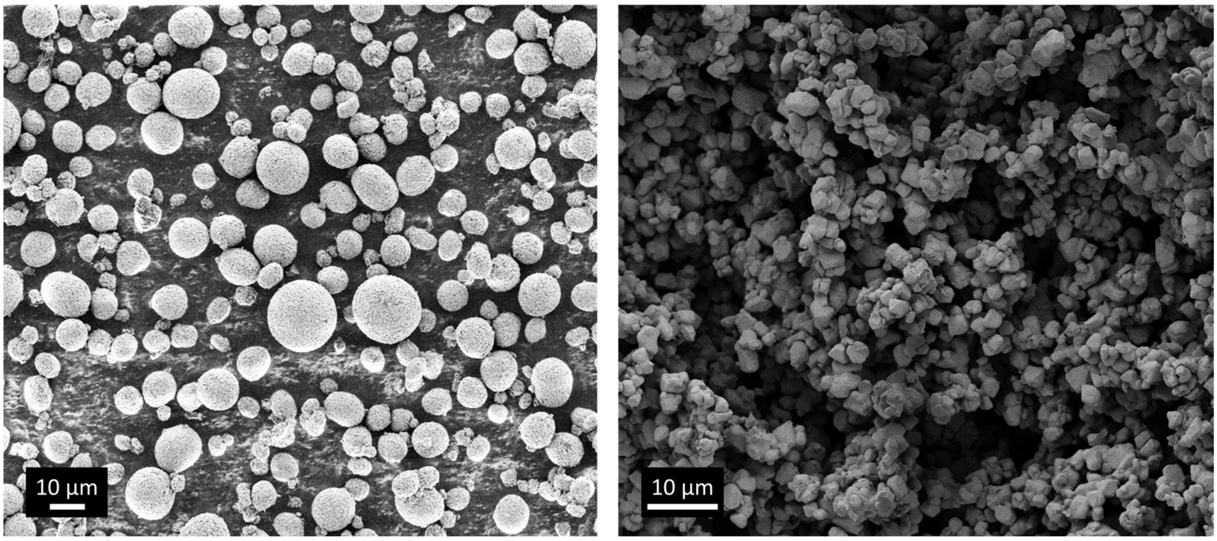

Figure S3. SEM micrographs of p-NMC811 (left) and s-NMC811 (right).

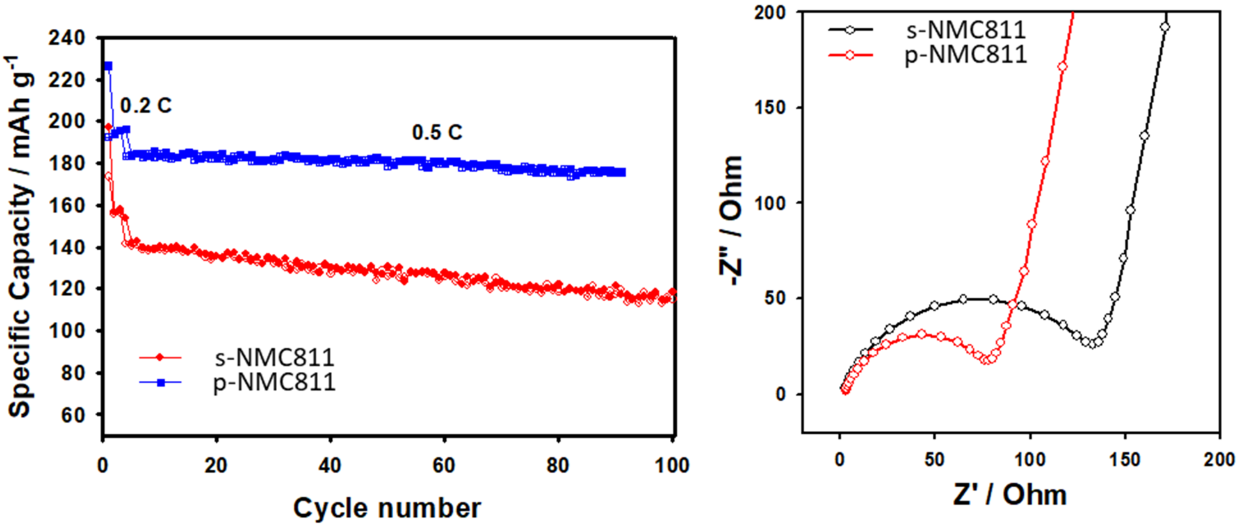

Figure S4. cycling performance of s-NMC811 and p-NMC811 in 1 M LiPF6 in EC/DEC 1:1 vol./vol.).

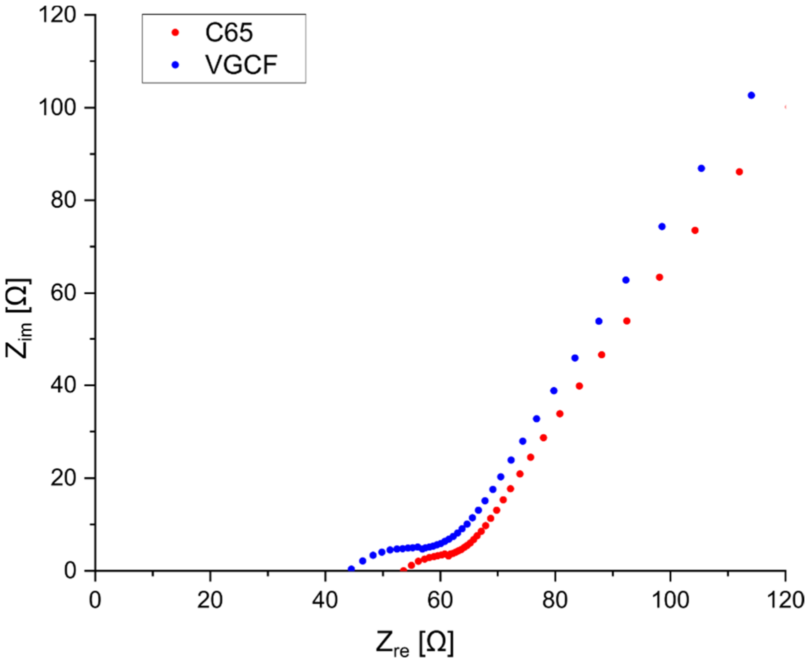

Figure S5. EIS data before cycling of electrodes, with C65 and VGCF as conductive additives.

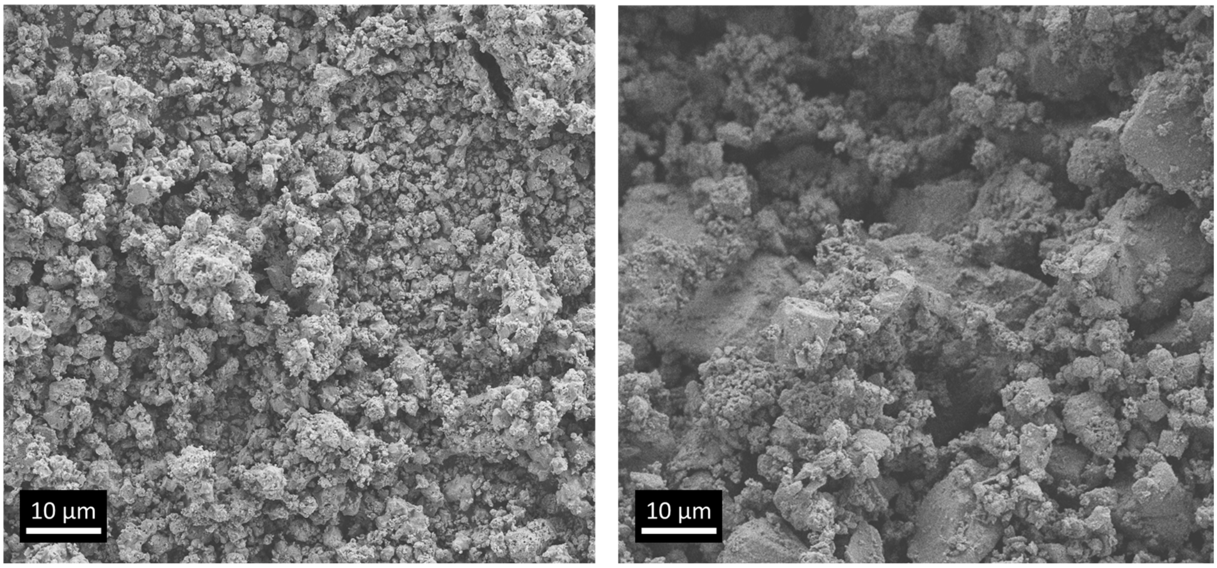

Figure S6. SEM micrographs of sp-Li_6PS_5Cl (left) and lp-Li_6PS_5Cl (right).

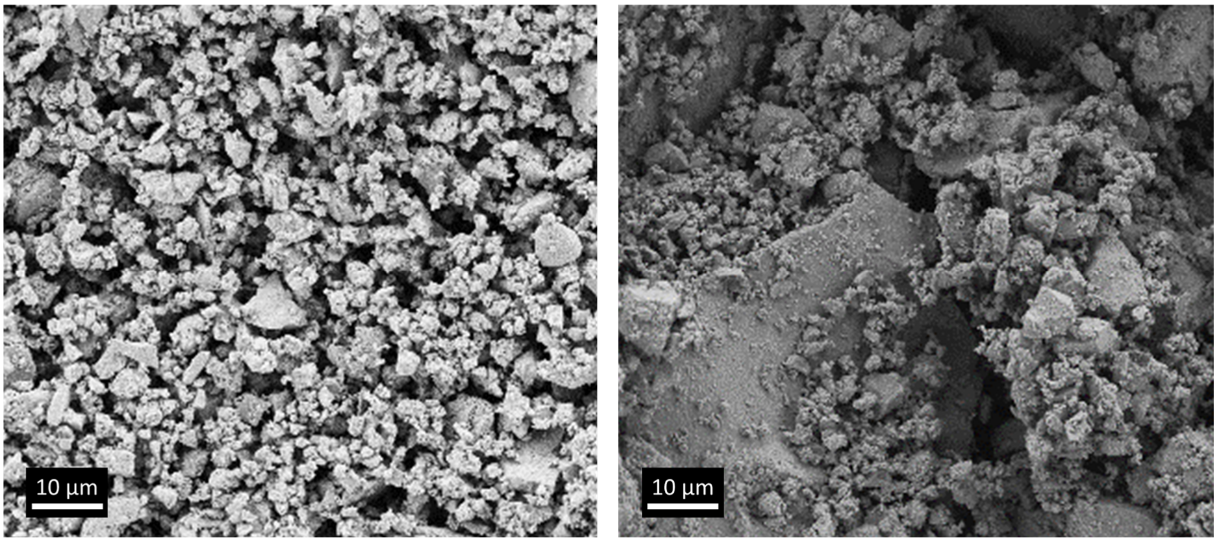

Figure S7. SEM micrographs of lp-Li_6PS_5Cl and lp-Li_6PS_5Cl (ball milled).

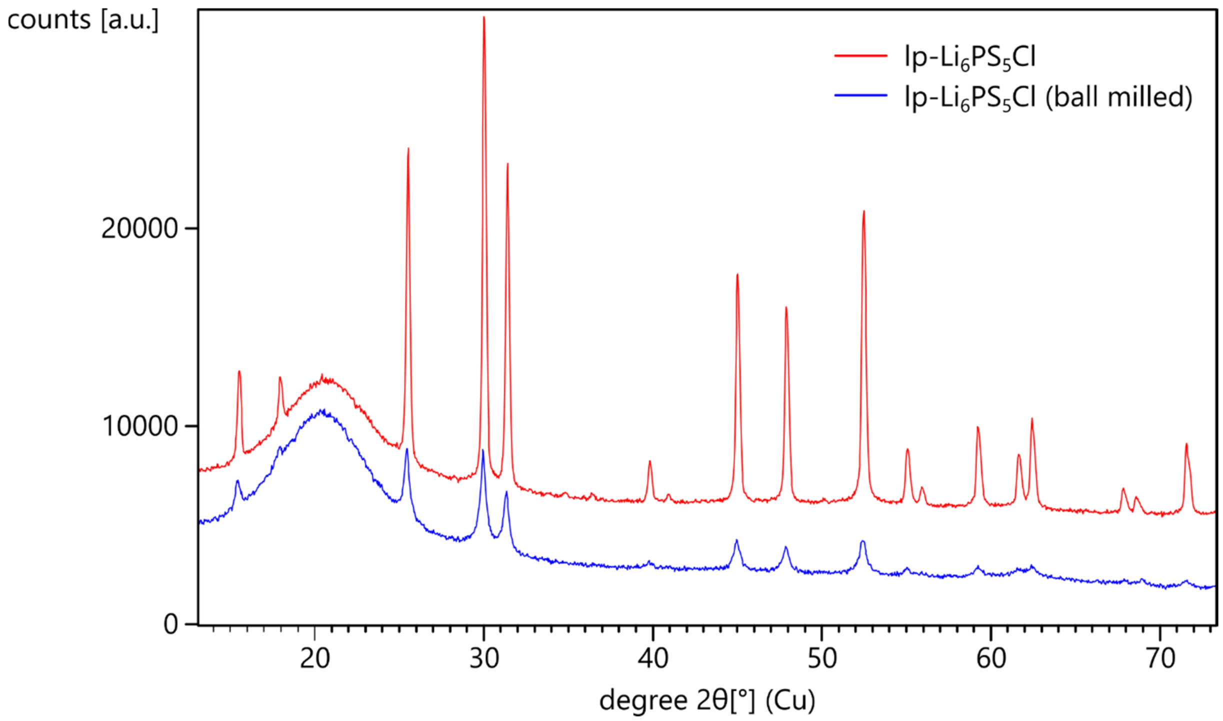

Figure S8. XRD pattern of lp-Li_6PS_5Cl and lp-Li_6PS_5Cl (ball milled).

Table S1. list of all composite cathode formulations used in this work.

	AM	SE	CA	BM	AM:CA:SE:BM [wt./wt.]	AM:CA:SE:BM [vol./vol.]	AM:SE [vol./vol.]
1	p-NMC811	sp-Li_6PS_5Cl	C65	-	67:3:30:0	40:5:55:0	42:58
2	s-NMC811	sp-Li_6PS_5Cl	C65	-	67:3:30:0	40:5:55:0	42:58
3	s-NMC811	sp-Li_6PS_5Cl	VGCF	-	67:3:30:0	40:5:55:0	42:58
4	s-NMC811	lp-Li_6PS_5Cl	C65	-	67:3:30:0	40:5:55:0	42:58
5	s-NMC811	lp-Li_6PS_5Cl (ball-milled)	C65	-	67:3:30:0	40:5:55:0	42:58
6	s-NMC811	sp-Li_6PS_5Cl	C65	-	64:3:33:0	38:5:57:0	40:60
7	s-NMC811	sp-Li_6PS_5Cl	C65	-	73:3:24:0	48:5:47:0	50:50
8	s-NMC811	sp-Li_6PS_5Cl	C65	-	79:3:18:0	57:5:38:0	60:40
9	s-NMC811	sp-Li_6PS_5Cl	C65	-	85:3:12:0	66:5:29:0	70:30
10	s-NMC811	sp-Li_6PS_5Cl	C65	-	90:3:7:0	77:5:18:0	80:20
11	s-NMC811	sp-Li_6PS_5Cl	C65	SBS	74.5:2.5:20.5:2.5	48:5:39:8	55:45
12	s-NMC811	sp-Li_6PS_5Cl	C65		76:3:21:0	52:5:43:0	55:45
13	s-NMC811	sp-Li_6PS_5Cl	C65		81:3:16	60:5:35	63:37
